# The effects of TNF-alpha inhibitor therapy on the incidence of infection in JIA children: a meta-analysis

**DOI:** 10.1186/s12969-019-0305-x

**Published:** 2019-01-18

**Authors:** Arnold Nagy, Péter Mátrai, Péter Hegyi, Hussain Alizadeh, Judit Bajor, László Czopf, Zoltán Gyöngyi, Zoltán Kiss, Katalin Márta, Mária Simon, Ágnes Lilla Szilágyi, Gábor Veres, Bernadett Mosdósi

**Affiliations:** 1 Department of Paediatrics, Medical School, University of Pécs, 7. József Attila street, Pécs, 7623 Hungary; 2 Institute of Bioanalysis, Medical School, University of Pécs, Pécs, Hungary; 3 Institute for Translational Medicine, Medical School, University of Pécs, Pécs, Hungary; 4 Division of Gastroenterology, First Department of Medicine, Medical School, University of Pécs, Pécs, Hungary; 5 Momentum Gastroenterology Multidisciplinary Research Group, Hungarian Academy of Sciences - University of Szeged, Szeged, Hungary; 6 Division of Haematology, First Department of Internal Medicine, Medical School, University of Pécs, Pécs, Hungary; 7 Division of Gastroenterology, First Department of Internal Medicine, Medical School, University of Pécs, Pécs, Hungary; 8 Division of Cardiology, First Department of Internal Medicine, Medical School, University of Pécs, Pécs, Hungary; 9 Department of Public Health Medicine, Medical School, University of Pécs, Pécs, Hungary; 10 First Department of Paediatrics, Semmelweis University, Budapest, Hungary; 11 Institute for Translational Medicine, Medical School, University of Pécs, Pécs, Hungary; 12 Szentágothai Research Centre, Medical School, University of Pécs, Pécs, Hungary; 13 Department of Psychiatry and Psychotherapy, Medical School, University of Pécs, Pécs, Hungary; 14 Institute of Surgical Research, University of Szeged, Szeged, Hungary; 15 Department of Paediatrics, Medical School, University of Debrecen, Debrecen, Hungary

**Keywords:** DMARD, Infection, JIA, Placebo, TNF-alpha inhibitor

## Abstract

**Background:**

Juvenile Idiopathic arthritis (JIA) is the most common chronic rheumatic disease in childhood. The diagnosis is based on the underlying symptoms of arthritis with an exclusion of other diseases Biologic agents are increasingly used on the side of disease-modifying anti-rheumatic drugs (DMARD) in JIA treatment.

**Main body:**

The aim of this meta-analysis was to investigate the observed infections in JIA children during tumor necrosis factor (TNF)-alpha inhibitor therapy. A systematic search of three databases (Medline via PubMed, Embase, Cochrane Library) was carried out up to May 2018. Published trials that evaluated the infectious adverse events in patients receiving TNF-alpha inhibitor vs. a control group were included in the analysis. Full-text data extraction was carried out independently by the investigators from ten relevant publications. 1434 patients received TNF-alpha inhibitor therapy; the control group consisted of 696 subjects. The analysis presented the risk of infection in the active treatment group (OR = 1.13; 95% CI: 0.76–1.69; *p* = 0.543). The majority of infections were upper respiratory tract infections (URTIs). Furthermore, the subgroup analysis demonstrated a higher infection rate in the observed localization.

**Conclusion:**

Anti-TNF therapy slightly but not significantly increases the incidence of infection in JIA children compared to other therapies (GRADE: moderate evidence). The most common infections reported were mild URTIs. Further studies with larger patients number with a strong evidence level are crucially needed to finalize the answer whether anti-TNF therapy elevates and if yes on what extent the incidence of infection in JIA children.

**Trial registration:**

Prospero: CRD42017067873.

**Electronic supplementary material:**

The online version of this article (10.1186/s12969-019-0305-x) contains supplementary material, which is available to authorized users.

## Background

JIA is the most common chronic inflammatory disease of unknown etiology in childhood. It is a heterogeneous autoimmune disease, falling into seven categories according to the International League of Associations for Rheumatology (ILAR) classification criteria [1]. This classification is based on the number of joints affected during the first six months of the disease and on the extra-articular involvements. The diagnosis is based on the clinical manifestations of inflamed joints with an exclusion of other diseases. Advances in the understanding of immunity and inflammation of the disease have led to novel therapies for treatment. Patients with JIA, who had partial response to synthetic DMARDs are treated with biologic agents, such as anti-TNF agents or IL-1- or IL-6- antagonists, or T-cell inhibitors [2]. TNF inhibitors were the first biologic disease-modifying anti-rheumatic drugs to be used for treating JIA. Two classes of TNF**-**alpha blocking agents are currently used in managing rheumatologic conditions: the monoclonal anti-TNF antibodies, such as infliximab (INX), adalimumab (ADA), golimumab, and certolizumab pegol, and the soluble TNF receptor, etanercept (ETA). They are recommended as second or third-line agents in the poly- or oligoarticular forms of JIA, following at least three months of DMARD therapy [2, 3]. The efficacy of anti-TNFs has been established in numerous trials. These drugs have been shown to improve symptoms, physical functioning, and quality of life [4–7]. Safety concerns for TNF inhibitors are primarily related to their immunosuppressive effects. Patients receiving biologics are generally at increased risk of certain viral and fungal infections, and opportunistic infections, or reactivation of mycobacterial infections [8–11]. In addition to the immunosuppressive effects of these agents, concomitant use of other immunosuppressive drugs, such as steroids or methotrexate (MTX), and the underlying inflammatory disease likely contribute to increased infectious risk [12–15]. The primary aim of this meta-analysis was to explore whether the TNF-alpha inhibitor therapy leads to an increased risk of infection in JIA children.

## Main text

To achieve the highest standard for systematic reviews and meta-analyses, the present study was developed according to the recommendations issued for the Preferred Reporting Items for Systematic Reviews and Meta-Analyses (PRISMA-P) protocols [16]. (PRISMA checklist. Additional file 1). RCTs or prospective comparative cohort studies were evaluated, the risk of bias and quality of evidence assessment was conducted using the JADAD and Newcastle-Ottawa Scale (NOS), and the quality of evidence was evaluated with the Grading of Recommendations Assessment, Development, and Evaluation (GRADE) system [17–19].

### Literature sources

A systematic search of the literature was carried out up to May 2018. The search included articles available in three different databases: in EMBASE, Medline via PubMed and the Cochrane Library.

### Strategy and study selection

Two reviewers manually conducted a comprehensive search with a combination of the following terms: juvenile AND idiopathic AND arthritis OR juvenile AND rheumatoid AND arthritis (using the old nomenclature) AND were crossed with etanercept OR adalimumab OR infliximab OR certolizumab (pegol) OR golimumab or tumor necrosis factor AND infection. Only English articles were screened, filters were used, if available, including human studies and age (< 18 years). As a result: 196 articles were found in EMBASE, 63 articles in PubMed and 34 in the Cochrane Library. The titles of and abstracts for the articles identified were assessed by the two reviewers. Therefore, only those prospective trials (with or without randomization) comparing infectious outcomes between a TNF-alpha inhibitor drug and placebo or DMARD therapy were eligible in the final result. Poster presentations, conference abstracts, case reports, retrospective studies and meta-analysis were rejected from the analysis. Studies using patient years were also excluded, since the data were impossible to combine statistically. Patient year expresses the incidents as the total number of events divided by the total follow up time. However, the length of exposure to the treatment is mostly different for different patients, and the patient year statistic cannot be calculated unless it is reported specifically. When the appropriateness of an article was in question, the full-text was evaluated. A third reviewer was consulted if required for a consensus. Data synthesis. The statistical analysis was conducted with Stata 11 SE (Stata Corp). The number of patients with observed infection in TNF-alpha inhibitor groups and control groups was used to calculate the odds ratio (OR). OR > 1 indicates the elevated risk of infection in the TNF-alpha inhibitor group compared to the control group. It reveals significant relationship if both CI > 1. ORs were pooled using the random effects model with the DerSimonien-Laird estimator and displayed on forest plots. To pool the specific infections, the Peto method was used, as recommended in the Cochrane Handbook for the meta-analysis of rare events [20]. Summary OR estimation, *p*-value and 95% Confidence Interval (CI) were calculated. *P* < 0.05 was considered a significant difference from summary OR = 1. Statistical heterogeneity was analyzed using the I2 statistic and the chi-square test to ascertain probability values; *p* < 0.05 was defined indicating significant heterogeneity.

### Outcome

This meta-analysis investigated prospective trials comparing infection risk in JIA children treated with TNF-alpha inhibitor, in contrast with JIA children receiving DMARD therapy, or placebo in certain publications. The studies that were included investigated four different biologic therapies of currently and previously licensed anti-TNF agents: ETA, ADA, INX and golimumab. The primary outcome was the odds of infection in the TNF-alpha inhibitor group compared to the control groups.

## Results

### Search results and study characteristics

**Fig. 1:**
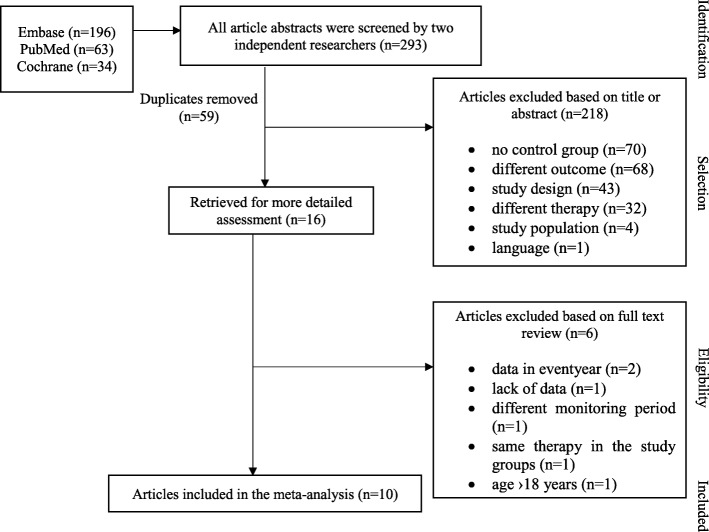
Flowchart of the articles included

Of the 293 publications, ten were eligible for the meta-analysis. 218 studies were rejected on the basis of not fulfilling the inclusion criteria (Fig. 1). After duplicates were removed, sixteen articles were retrieved for more detailed analysis. Of the sixteen studies, further exclusions were made: one was discounted because of lack of data [21]. Ruperto et al. was rejected due to the difference between the follow-up period of the studied groups [7]. Two articles were excluded owing to the data only being available in patient years, resulting in numbers being impossible to combine statistically [6, 22]. One article compared ETA and MTX treatment with an ETA only cohort. Since both study populations received anti-TNF therapy, it was not possible to compare the infectious adverse events [23]. Walters et al. was excluded due to 18–21 year old patients participating [24]. Eventually, ten trials proved appropriate for the final assessment. Data extraction from full-length articles was conducted independently by the two researchers.

No distinction was made between JIA-categories due to the small number of trials available in the field. Studies evaluating systemic onset JIA were few [25–27]. The research mainly consisted of oligo- and polyarticular forms of JIA. [25–31] Subjects with psoriatic and enthesitis-related arthritis categories were furthermore presented in the analysis. [25, 26, 28, 29, 32, 33] In two studies TNF-alpha inhibitor therapy was initiated because of active uveitis associated with JIA [28, 34]. Studies were heterogeneous in the observational period, drug types, drug dosing, disease activity index, and mean length of disease at enrolment. In the case of a study consisting of two parts, e.g. a twelve-week open-label lead-in phase and a twelve-week double-blind, randomized controlled phase, only the latter was included in the analysis, since the former did not contain a control group, as discussed above. Table 1 shows the study baseline characteristics.

**Table 1:** Baseline characteristics of the trials included

Source	Patient Number (n)	Drug Type (No. of Participants)	Control Type (No. of Participants)	JIA Category	Study Type	Age (years)	Length of follow-up (months)	Risk of Bias	Disease duration TNFi (ys)	Disease duration control (ys)	Active joints with arthritis TNFi	Active joints with arthritis control	Physician’s global assessment of disease activity (0–10) TNFi	Physician’s global assessment of disease activity (0–10) control	Concomitant therapy – TNFi N (%)	Concomitant therapy - control: N (%)	GC - TNFi	GC - control	Comorbidity TNFi	Comorbidity control	Previous treatment (TNFi + control)
Burgos-Vargas et al. 2015	46	Adalimumab [31]	Placebo [15]	ERA	RCT, DB	6–18	12	JADAD 4	2.6 ± 2.3	2.7 ± 2.5	8.4 ± 7.1	6.7 ± 5.3	5.3 ± 2.2	5.2 ± 20.5	27 (87.1) NSAID 21 (67.7) DMARD	14 (93.3) NSAID 11 (73.3) DMARD	N = no data 0.2 mg/kg	N = no data 0.2 mg/kg	0	0	NSAID, DMARD, GC
Horneff et al. 2015	38	Etanercept [20]	Placebo [18]	ERA	RCT, DB	6–18	6	JADAD 3	2.4 ± 2.1	3.2 ± 3.5	5.7 ± 2.6	5 ± 2.6	5.2 ± 1.9	5.2 ± 1.8	NSAID 12 (60.0) SSZ	NSAID 14 (77.7) SSZ	N = no data 0.2 mg/kg	N = no data 0.2 mg/kg	0	0	NSAID, DMARD, GC, etanercept
Brunner et al. 2018	154	Golimumab (78)	Placebo (76)	PA, OA, SOJIA, Psoriatic	RCT, DB	2–17	12	JADAD 4	> 6 mo	> 6 mo	14.8 ± 9.2	15.0 ± 10.6	5.5 ± 2.0	5.7 ± 1.8	NSAID 78 (100) MTX	NSAID 76 (100) MTX	*N* = 19 0.2 mg/kg	*N* = 14 0.2 mg/kg	0	0	NSAID, MTX, GC Golimumab
Wallace et al. 2012	85	Etanercept [42]	Placebo [43]	PA	RCT, DB	2–17	12	JADAD 4	4.9 ± 0.5 mo	5.2 ± 0.6 mo	18.3 ± 11.0	25.5 ± 14.4	7.0 ± 1.8	7.1 ± 1.9	42 (100) MTX	43 (100) MTX	*N* = 42 0.5 mg/kg/d	*N* = 0	0	0	MTX, GC
Giannini et al. 2009	491	Etanercept (294)	DMARD (197)	PA, OA, SOJIA	Prospective cohort, non-randomized	2–18	36	NOS 8	40.7 ± 41.7 mo	20.2 ± 30.7 mo	6	6	4	4	249 (84.7) NSAID 294 (100.0) MTX	180 (91.4) NSAID 197 (100.0) MTX	*N* = 78	*N* = 36	*N* = 2 Uveitis	0	NSAID, DMARD, GC, etanercept
Smith et al. 2005	12	Etanercept [7]	Placebo [5]	JIA	RCT, DB	2–18	6	JADAD 5	No data	No data	No data	No data	No data	No data	3 (42.8) MTX	4 (80.0) MTX	N = 2	N = 2	N = 7 Uveitis	*N* = 5 Uveitis	MTX, GC
Tynjala et al. 2011	40	Infliximab [20]	DMARD [20]	PA	Prospective cohort, randomized	4–15	12,5	NOS 7	1.5 ± 0.3 mo	1.8 ± 1.1 mo	18 ± 10.0	18 ± 12	4.9 ± 1.8	6 ± 1.8	NSAID 20 (100) MTX	NSAID 20 (100) MTX	N = 2 0.1 mg/kg	N = 0	0	N = 2 Uveitis	No previous systemic therapy
Ramanan et al. 2017	90	Adalimumab (60)	Placebo [30]	PA, OA, Psoriatic	RCT, DB	2–18	24	JADAD 5	5.58 ± 3.69	4.81 ± 3.19	0	0	0.7 ± 1.4	0.83 ± 1.09	60 (100) MTX	30 (100) MTX	N = 2 0.14 mg/kg	N = 0	*N* = 60 uveitis	*N* = 30 uveitis	MTX, GC
Davies et al. 2015	1112	Etanercept (852)	DMARD (260)	PA, OA, ERA SOJIA, Psoriatic Unspecified	Prospective cohort, registry	4–17	Med. 34	NOS 8	3	1	5	6	3.5	4.0	453 (53) MTX	260 (100) MTX	*N* = 184	*N* = 47	*N* = 85 uveitis	*N* = 26 uveitis	MTX, GC
Muller et al. 2017	62	Etanercept [30]	DMARD [32]	PA, OA, Psoriatic	RCT, SB	2–16	3	JADAD 3	8.5 mo	7.8 mo	5.1	4.8	8.5	7.5	NSAID 30 (100) MTX	NSAID 32 (100) MTX or SSZ	N = 3	N = 0	0	0	NSAID

### Patients

Overall, the study population consisted of 2130 patients. 1434 subjects received at least one dose of TNF-alpha inhibitor, and 696 patients were selected to receive DMARD therapy or a placebo as a control group. The meta-analysis involved those patients who were part the safety analysis. Therefore, patients withdrawn before the safety assessment for the study were not included in the final population of 2130. Within the active agent group, 20 patients were treated with intravenous infliximab, 78 with golimumab, 1245 with subcutaneous ETA and 91 with subcutaneous ADA as a TNF-alpha inhibitor. Concomitant drug therapy consisted of DMARDs and NSAIDs as systemic treatments. Low dose glucocorticoids (< 0.2 mg/kg prednisone equivalent or < 10 mg/day, whichever was less) were also permitted. Prior treatment with biologic was presented in three studies [26, 27, 33].

### Control groups

Two types of control groups were used in the studies. There were seven publications with an RCT design, six using a placebo [26, 28, 31–34] and one using DMARD [29] as a control group. However, in every single placebo-controlled trial, the patients received concomitant DMARD therapy in both (active and control) groups, which was MTX [26, 28, 31, 34] or Sulphasalazine (SSZ) [33]. One study permitted both MTX and SSZ (MTX/SSZ) [32]. The other control group underwent DMARD alone. One RCT [29] and three prospective cohort studies [25, 27, 30] compared the safety of a TNF antagonist with DMARD therapy. In the trials, the DMARD therapy involved either MTX [25, 27, 30] or MTX/SSZ [29].

### Risk of bias, and quality of evidence assessment

The JADAD scale was used to evaluate RCTs [17]. All RCTs were assigned points from one to five for randomization, blinding procedure and an account of all patients (Table 1). Each study received at least three points; no poor-quality articles therefore remained in the analysis. The JADAD scale is commonly used for evaluating randomized, controlled trials. It is easy to use, reliable and valid. The maximum of two points could be calculated for randomization, also two points for adequate blinding procedure and one extra point for clear data presentation. In the cases of prospective cohort studies, the risk of bias was assessed using the Newcastle-Ottawa Scale (NOS) [18]. Table 1 summarizes the risk of bias in the non-RCTs as well. The NOS evaluates the risk of bias in individual studies throughout assessing selection, comparability and outcome. For selection a maximum of four points-, for comparability a maximum two points can be awarded. There are three items measuring the outcome, therefore a maximum of three points can be calculated. The more points a study was collected, the lesser risk of bias it has (Additional file 2).

The authors estimated the quality of evidence of this meta-analysis as moderate based on the Grading of Recommendations, Assessment, Development, and Evaluation (GRADE) system [19]. The GRADE system is a widely used method to assess the studies that are involved in systematic reviews and meta-analysis. Based on it, recommendations could be formed. After ranking and upgrading or downgrading the trials as step one and two a final grade can be assigned for each outcome. This can be high, moderate, low or very low grade. In step four and five a recommendation is to be made. In this meta-analysis the trials included were RCTs and well-designed prospective cohort studies with transparent outcomes and recommendations; the assessment of individual study validity thus revealed no potential sources of high risk of bias, based on measurements using the NOS or JADAD scale.

### Outcome: Infectious adverse events

**Fig. 2:**
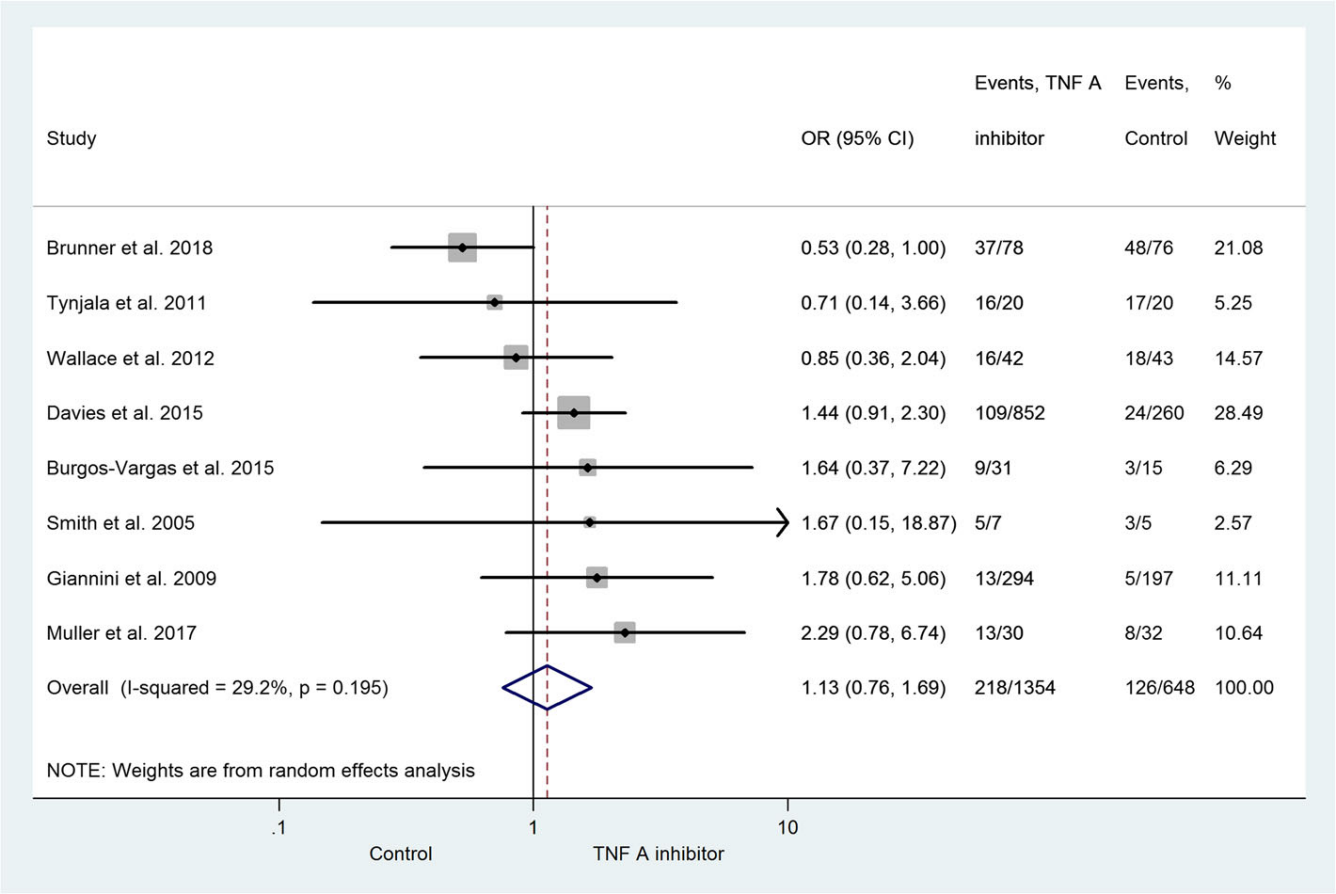
The risk of infection between the TNF-alpha inhibitor and the non-TNF-alpha inhibitor group. The size of the grey marker is proportional to the weight of the study. (OR = 1.13; 95% CI: 0.76–1.69; *p* = 0.543)

Eight trials reported 218 subjects with an infectious adverse event. Two trials contained no data on the total number of patients with infections. [28, 33] However, they included data on subgroups of patients with specific infections. Consequently, these two studies are only represented in the subgroup analysis. In the control group, 126 subjects developed some sort of infection. Figure 2 represents the infections developed during the monitoring period. The risk of infection was increased in the subjects receiving active therapy (OR = 1.13; 95% CI: 0.76–1.69; *p* = 0.543). A statistically significant risk of infection in the active treatment group was not demonstrated. Due to the small number of adequate studies in the field, it was not possible to set up a comparison between the reported anti-TNF drugs.

### Subgroup analysis

**Fig. 3:**
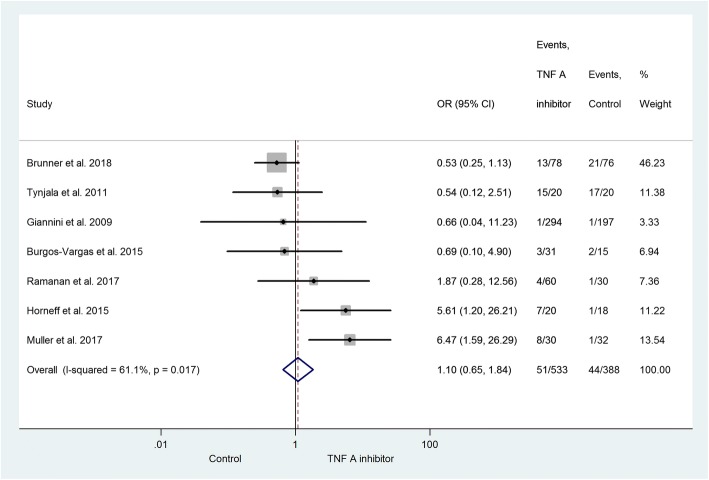
Forest plot on the occurrence of upper respiratory tract infection in the TNF-alpha inhibitor group vs. the control group. The risk of URTI is elevated in the former group (OR = 1.10; 95% CI: 0.65–1.84; *p* = 0.729)

**Fig. 4:**
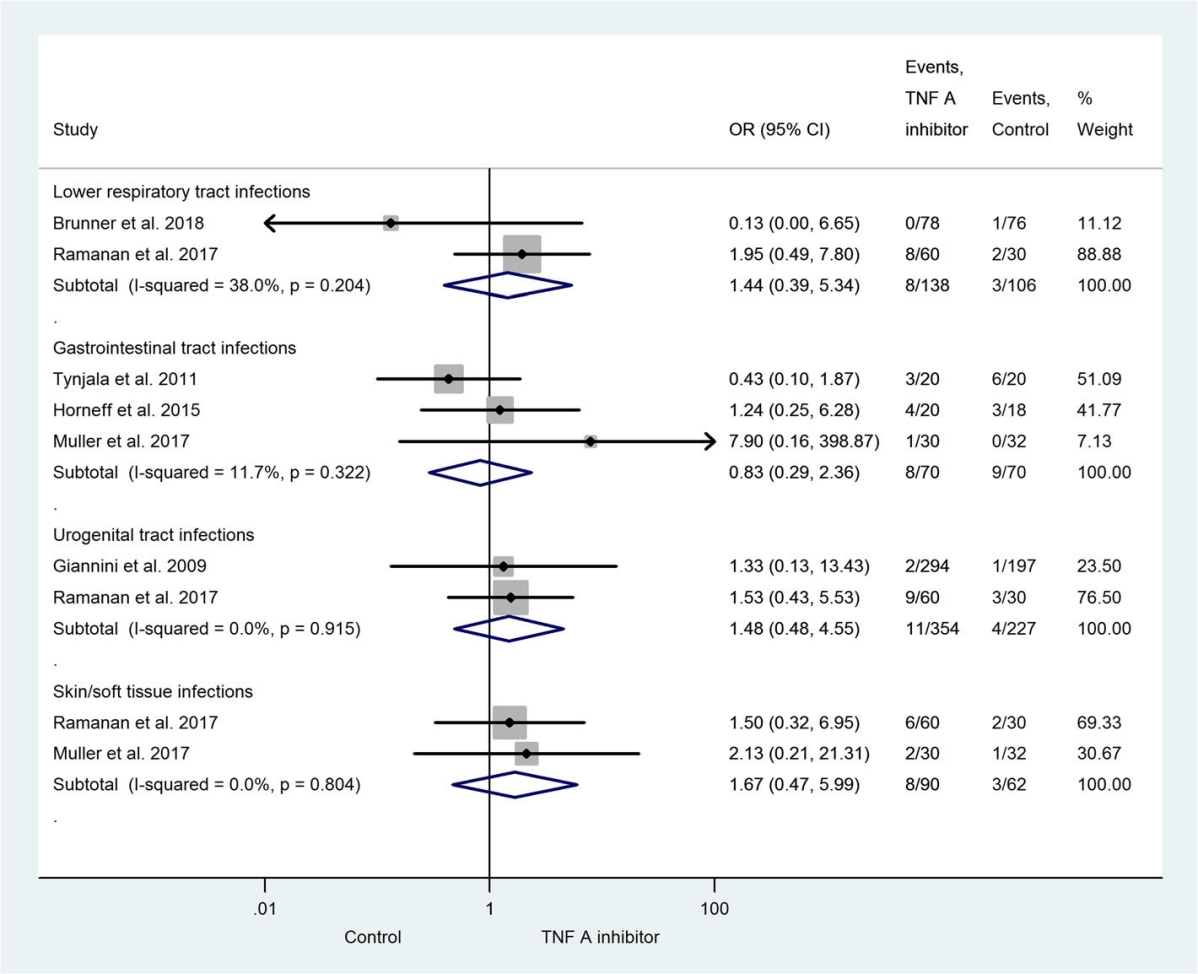
Effect of the TNF-alpha inhibitor group vs. the control group on the occurrence of the infectious diseases demonstrated. An increase (with exception of gastrointestinal tract infections) was observed in the risk of a particular infection in the TNF-alpha inhibitor group

The subgroup analysis consists of trials providing data on different types of infection. The most common infection was upper respiratory tract infection (URTI, Fig. 3) [26–30, 32, 33]. The risk of URTI is not significantly elevated in the active group (OR = 1.10; 95% CI: 0.65–1.84; *p* = 0.729). Among those articles with data on overall infection and URTI together, 23% URTI was found in the active treatment and 34% in the control group. Gastrointestinal tract infections were presented in three studies with an OR of 0.83 (95% CI: 0.29–2.36; *p* = 0.721) [29, 30, 33]. The statistical analysis also included lower respiratory tract infections [26, 28] (OR = 1.44; 95% CI: 0.39–5.34; *p* = 0.581), urogenital tract infections [27, 28] (OR = 1.48; 95% CI: 0.48–4.55; *p* = 0.491), and skin and soft tissue infections [28, 29] (OR = 1.67; 95% CI: 0.47–5.99; *p* = 0.429). With an exception of gastrointestinal tract infections, the risk of developing these infections is elevated in patients treated with TNF-alpha inhibitor, but the relationship is not significant (Fig. 4). There were less gastrointestinal tract infections observed in the TNF-alpha inhibitor group. One patient in the active treatment group developed septicemia, but no patient in the control group did so [27]. Severe opportunistic infections did not present in the population under examination. Herpes zoster infections presented and two cases of localized moniliasis occurred, but no differences were observed between the groups under investigation (data not presented).

A random effect meta-regression was performed to investigate the effect of the study length on the association with an elevated risk of infections; the coefficient and the corresponding *p*-value was reported. There is a positive relationship between the OR and the length of the study, however it is not statistically significant. (Additional file 3: Figure S1). The small-study effect was tested with Egger’s test, with *p* < 0.05 indicating proof of bias (Additional file 4: Figure S2). A sensitivity analysis was also carried out omitting one study and calculating summary OR and 95% CI to investigate the influence of a single study on the final estimation (Additional file 5: Figure S3). The measure of inconsistency between trials (I^2^) was 29.2% (*p* = 0.195), indicating that the studies were not statistically heterogeneous.

### Tuberculosis (TB)

TB-screening is one of the preliminary tests before the induction of a biologic agent therapy. One trial that was excluded used a retrospective observational study design to investigate JIA patients with TB [35]. Latent TB infection prior to therapy was diagnosed in 3/221 adolescent girls (prevalence rate: 1.4%; 95% CI: 0.4–4.2). In this meta-analysis there was no patient diagnosed with TB during the monitoring period.

### Serious infections

In general, the definition of serious infection is an event that could be life threatening, requiring hospitalization, need of intravenous treatment, or associated with death. Adult population studies showed an increased rate of serious infections associated with TNF-alpha inhibitors [9, 36]. However, there are few studies investigating these infections in JIA-patients. The most common serious infection was pneumonia requiring hospitalization, with a low number of occurrence (one patient in the TNF-alpha group, two in the control group). Urosepsis with unknown pathogen occurred in one patient in the active treatment group. Therefore, this meta-analysis showed similar severe infectious events across the two study groups.

## Discussion

To our knowledge, this is the first meta-analysis to investigate the infectious adverse events in JIA children treated with TNF-alpha inhibitor. Infections are the most frequent diseases in childhood. The general risk factors for recurrent or severe infections could be conditions such as primary, secondary, or acquired immunodeficiency.

The meta-analysis confirmed that anti-TNF therapy slightly but not significantly increases the incidence of overall infection compared to non-biological therapies. Since the association was not significant, it could be either a real effect or a coincidence only. The risk of infection appears to be increased in JIA patients as a result of the disease itself. Generally, patients treated with TNF-alpha inhibitors are characterized by a longer disease duration and higher disease activity [15]. Immunosuppressive therapies such as anti-rheumatic drugs, elevate the risk even more [37–39]. Horneff reported a link between moderate dose of corticosteroid and increased risk of infection [40].

However, the results demonstrated that the most common infectious events reported in JIA patients were mild URTIs (23% in the TNF-alpha inhibitor group, 34% in the control group), which are also widely represented in the healthy population [41–43].

Additionally, our investigation pointed out that there was no significant difference between the TNF-alpha inhibitor and the control group regarding the incidence of infection of lower respiratory tract, gastrointestinal tract, urogenital tract and soft tissue. The incidence of serious infections is low throughout all clinical trials performed in JIA patients [15, 40]. Our study was not able to compare the exact incidence of serious infections due to the incongruent definition across studies and the small number of patients with severe infections.

In adult population, Askling et al. recognized greater risk of infection during the first six months of anti-TNF therapy, with a decrease over time [44]. Time-varying risk of infection was only reported in one study, therefore it could not be statistically analyzed [25]. The microbiologic results of mild and severe infections in the patients were limited; most of the studies supplied no information on the severity of the infections or on the pathogens involved. Herpes virus was the most commonly identified viral pathogen in the articles. Other than varicella zoster and two cases of moniliasis, opportunistic pathogens have not been found. Therefore, the relation between opportunistic infections and TNF antagonists could not be demonstrated in this analysis.

The other main concern with biological treatment is TB [45, 46]. In this investigation there was no subject diagnosed with TB during the study period. Also, the authors would like to emphasize the importance of fungal infections. The most frequent invasive mycotic agent is histoplasma. The clinical features are similar to those which are seen in acute TB. Patients on immunosuppressive treatment are at increased risk of developing disseminated histoplasmosis leading to high rate of mortality [47–50]. However, there was no data found regarding histoplasma infections among the reported articles. In this meta-analysis, relation between elevated risk of TB, histoplasmosis and anti-TNF drugs, could not be found.

Furthermore, the authors also wish to point out some important limitations of the study. Experience from clinical trials investigating paediatric population are often limited due to low number of patient. The included trials were clinically heterogeneous in terms of JIA category, disease duration, previous and concomitant drugs used, and infection interpretation. The papers principally investigated ETA as an active treatment. There were only few studies investigated ADA, INX or golimumab. Therefore, no comparison was set up between the TNF-alpha inhibitor drugs.

A major limitation of this meta-analysis is the meagre evidence published to date. Most of the articles have short follow-up period, resulting in limited power to detect rare events. Further studies with strong evidence and longer monitoring period are called for examine the pathogens involved in the infections, the precise severity of the infectious diseases and the localizations as well as to compare the different kinds of TNF-alpha inhibitor drugs.

## Conclusion

Biologics in combination with other immunosuppressive agents, such as MTX and corticosteroids, have become an important component in the effective management of patients in the pediatric population with a variety of autoimmune conditions, such as JIA. The benefits of treatment with biologics in the past two decades outweigh the possible risk of infection. This meta-analysis demonstrates that anti-TNF-alpha therapy slightly but not significantly increases the incidence of infection compared to other therapies in JIA children (GRADE, moderate evidence). The number of serious and opportunistic infections was low. Data on the long-term safety of anti-TNFs and other biologics in children and adolescents are still scarce.

## Additional files

Additional file 1: PRISMA-Checklist. (DOC 65 kb)

Additional file 2: Risk of bias. This work is dedicated to the 650th anniversary of the University of Pécs. (DOCX 56 kb)

Additional file 3: **Figure S1.** Meta-regression. There is a positive relationship (coefficient = 0.0035507 *p* = 0.374 between the OR and the length of the study, however it is not statistically significant. (TIF 34 kb)

Additional file 4: **Figure S2.** Begg’s funnel plot. The funnel plot demonstrates overall infections with regard to the small study effect. The dots representing the studies are located symmetrically around the point-estimate. There is no sign of the small study effect. The *p*-value arrived at with Egger’s test is 0.788, which supports the same finding. (TIF 26 kb)

Additional file 5: **Figure S3.** One study omitted analysis. An overall OR and 95% CI are shown next to the names of the authors, demonstrating a result without the article in question. The elevated risk of infection in the anti-TNF group does not change, if we exclude any of the articles. (TIF 54 kb)

## Abbreviations

ADA: Adalimumab
CI: Confidence Interval
DMARD: Disease-Modifying Anti-Rheumatic Drug
ETA: Etanercept
INX: Infliximab
JIA: Juvenile Idiopathic Arthritis
MTX: Methotrexate
NOS: Newcastle Ottawa Scale
NSAID: Non-steroid Anti-inflammatory Drug
OR: Odds Ratio
RCTs: Randomized Controlled Trials
SSZ: Sulphasalazine
TB: Tuberculosis
TNF-alpha: Tumor Necrosis Factor Alpha;
URTI: Upper Respiratory Tract Infection
