# Supplementary material for: The effects of TNF-alpha inhibitor therapy on the incidence of infection in JIA children: a meta-analysis
Source: Pediatr Rheumatol Online J. 2019 Jan 18;17:4. doi: 10.1186/s12969-019-0305-x (PMC6339290; doi:10.1186/s12969-019-0305-x)
Supplement: Supplementary file 2 — Risk of bias. This work is dedicated to the 650th anniversary of the University of Pécs. (DOCX 56 kb) [file 12969_2019_305_MOESM2_ESM.docx]

JADAD-scale (17)

| Study | Randomization is mentioned | Appropriate randomization | Blinding is mentioned | Appropriate blinding procedure | Follow-up on all patients is known | Result |
| --- | --- | --- | --- | --- | --- | --- |
| Burgos-Vargas et al. 2015 | 1 | 1 | 1 | 0 | 1 | 4 |
| Horneff et al. 2015 | 1 | 0 | 1 | 0 | 1 | 3 |
| Brunner et al. 2018 | 1 | 1 | 1 | 0 | 1 | 4 |
| Wallace et al. 2012 | 1 | 1 | 1 | 0 | 1 | 4 |
| Smith et al. 2005 | 1 | 1 | 1 | 1 | 1 | 5 |
| Ramanan et al. 2017 | 1 | 1 | 1 | 1 | 1 | 5 |
| Muller et al. 2017 | 1 | 0 | 1 | 0 | 1 | 3 |

**Figure 6.** Quality assessment of prospective cohort studies based on the Newcastle-Ottawa scale (18)

| References | Selection  1 2 3 4 | Comparability  1 | Outcome assessment  1 2 3 | Quality judgement |
| --- | --- | --- | --- | --- |
| Giannini et al | * * * * | * * | * * | * * * * * * * * |
| Tynjala et al | * * * * | * | * * | * * * * * * * |
| Davies et al | * * * * | * | * * * | * * * * * * * * |

*Note: A study can be awarded a maximum of one star for each numbered item within the Selection and Outcome categories. A maximum of two stars can be given for Comparability. Selection: 1) Representativeness of the exposed cohort (truly representative of the average or somewhat representative of the average in the community. 2) Selection of the non-exposed cohort (drawn from the same community as the exposed cohort). 3) Ascertainment of exposure (secure record (e.g. surgical records) or structured interview). 4) Demonstration that outcome of interest was not present at start of study (yes). Comparability: Comparability of cohorts on the basis of the design or analysis, a second star could be added if study controls for any additional factor. Outcome: 1) Assessment of outcome (independent blind assessment or record linkage). 2) Was follow-up long enough for outcomes to occur (yes). 3) Adequacy of follow up of cohorts (complete follow up - all subjects accounted for or subjects lost to follow up unlikely to introduce bias - small number lost).*

References:

17. Jadad AR, Moore RA, Carroll D, Jenkinson C, Reynolds DJ, Gavaghan DJ, et al. Assessing the quality of reports of randomized clinical trials: is blinding necessary? Control Clin Trials. 1996;17(1):1-12.

18. Hartling L, Hamm M, Milne A ea. Validity and Inter-Rater Reliability Testing of Quality Assessment Instruments [Internet]. Appendix E, Decision Rules for Application of the Newcastle-Ottawa Scale.: Rockville (MD): Agency for Healthcare Research and Quality (US). 2012 Mar.
