# Supplementary figures and images for: The effects of TNF-alpha inhibitor therapy on the incidence of infection in JIA children: a meta-analysis
Source: Pediatr Rheumatol Online J. 2019 Jan 18;17:4. doi: 10.1186/s12969-019-0305-x (PMC6339290; doi:10.1186/s12969-019-0305-x)

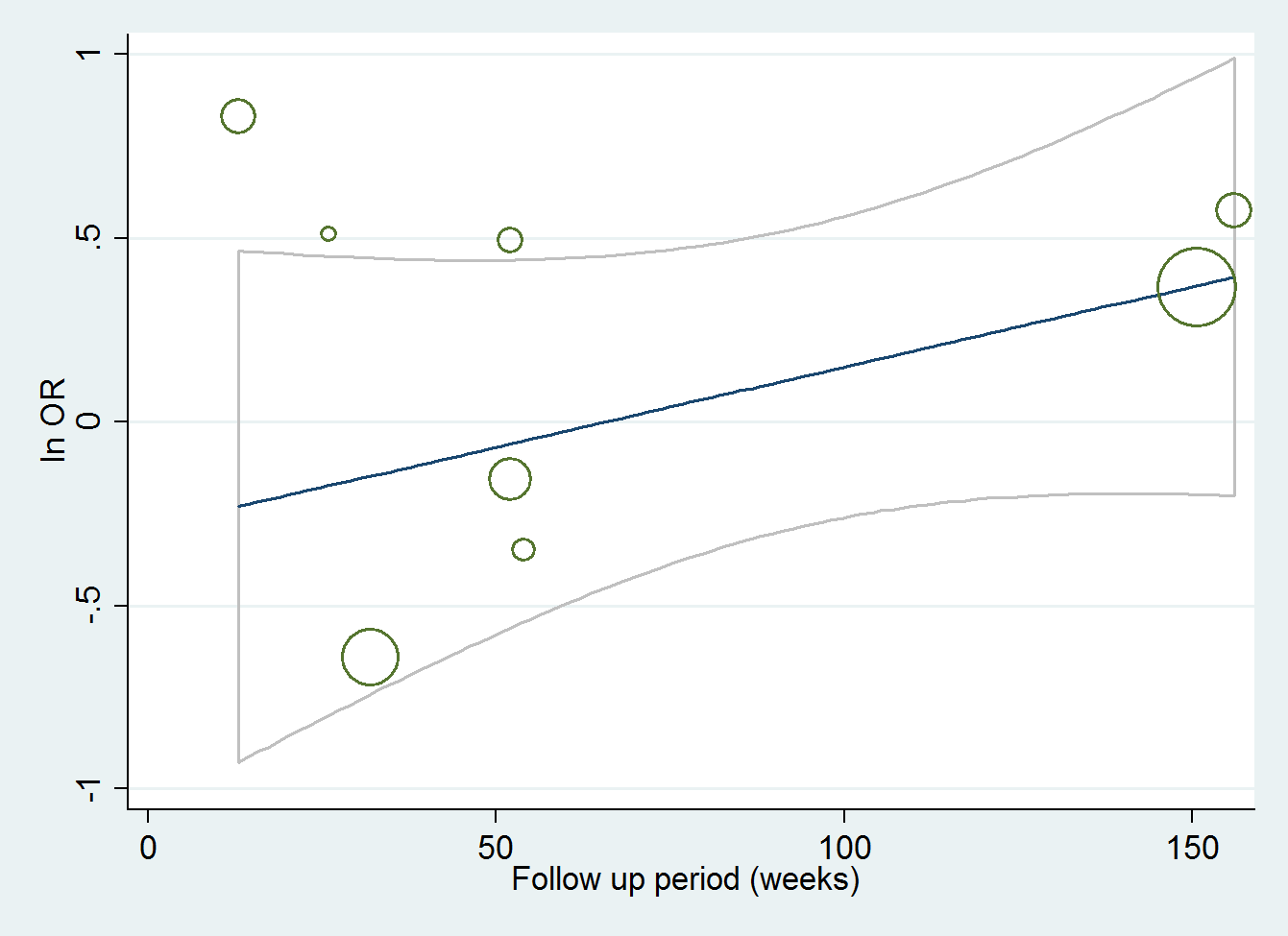

Supplement: Supplementary file 3 — Figure S1. Meta-regression. There is a positive relationship (coefficient = 0.0035507 p = 0.374 between the OR and the length of the study, however it is not statistically significant. (TIF 34 kb) [file 12969_2019_305_MOESM3_ESM.tif]

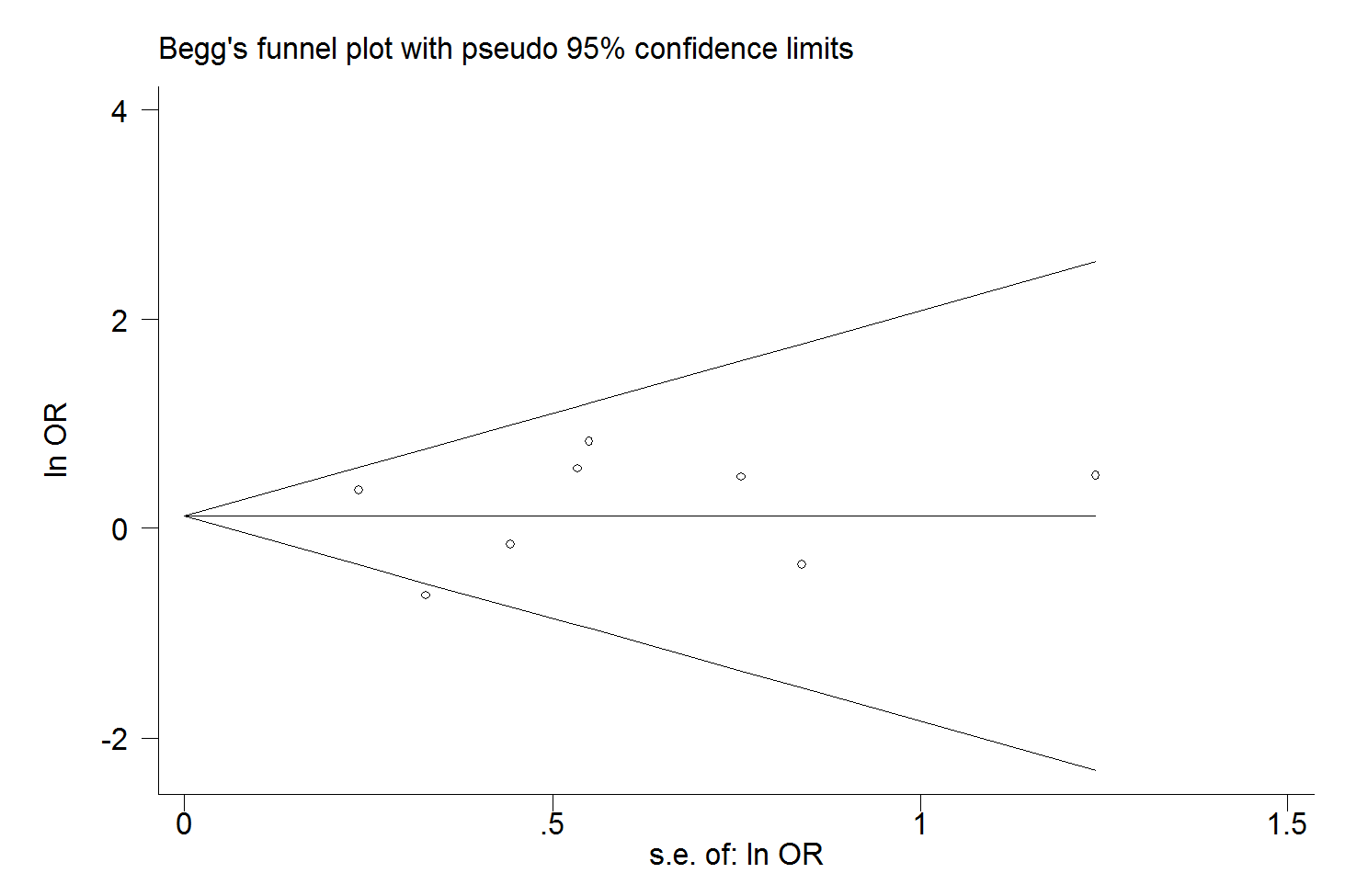

Supplement: Supplementary file 4 — Figure S2. Begg’s funnel plot. The funnel plot demonstrates overall infections with regard to the small study effect. The dots representing the studies are located symmetrically around the point-estimate. There is no sign of the small study effect. The p-value arrived at with Egger’s test is 0.788, which supports the same finding. (TIF 26 kb) [file 12969_2019_305_MOESM4_ESM.tif]

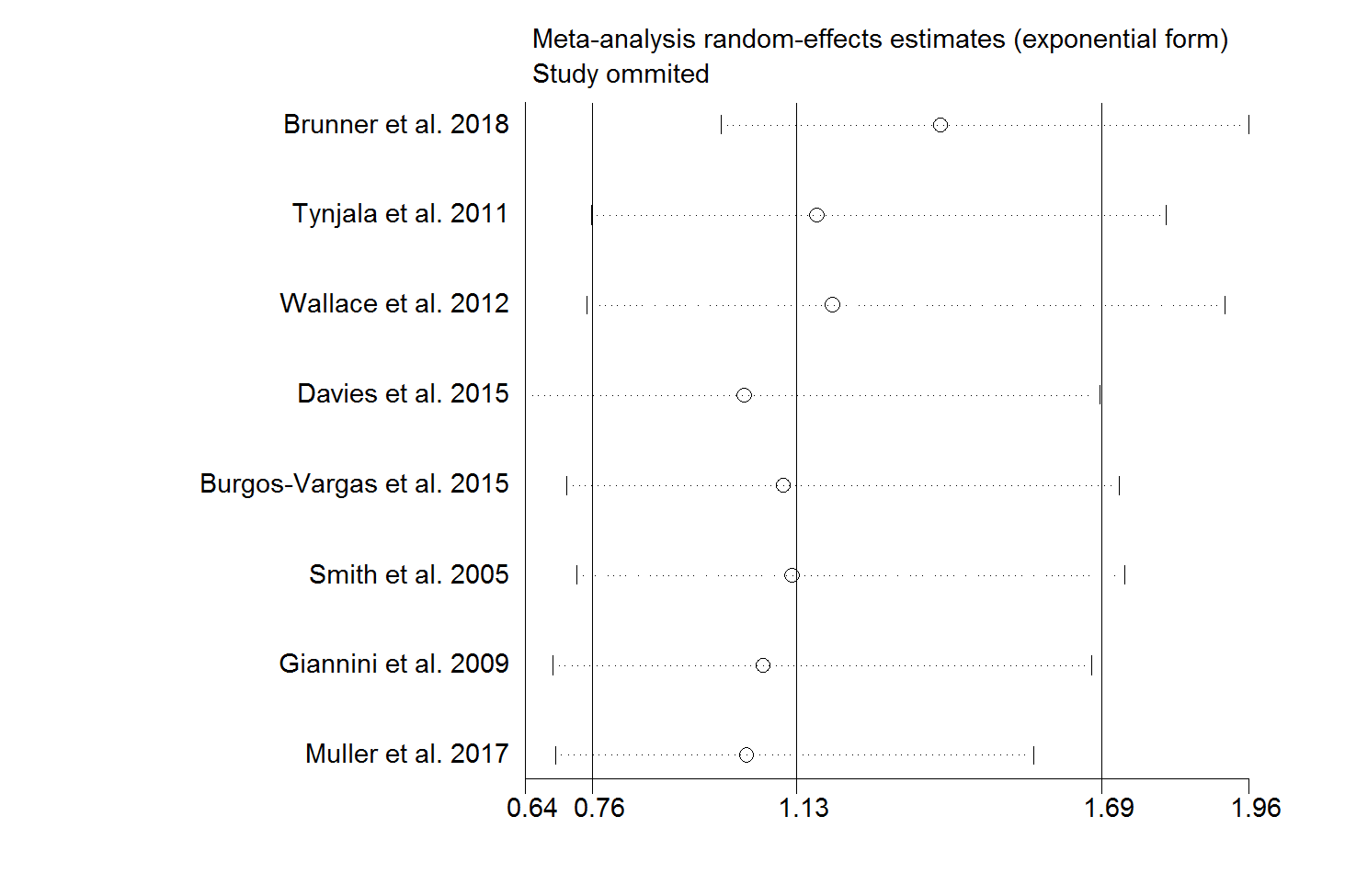

Supplement: Supplementary file 5 — Figure S3. One study omitted analysis. An overall OR and 95% CI are shown next to the names of the authors, demonstrating a result without the article in question. The elevated risk of infection in the anti-TNF group does not change, if we exclude any of the articles. (TIF 54 kb) [file 12969_2019_305_MOESM5_ESM.tif]
